# The ulnar digital artery perforator flap: A new flap for little finger reconstruction - A preliminary report

**DOI:** 10.4103/0970-0358.73445

**Published:** 2010

**Authors:** Nikhil Panse, Parag Sahasrabudhe

**Affiliations:** Department of Plastic Surgery, B.J. Medical College and Sasoon Hospital, Pune, India

**Keywords:** B.J. Flap, little finger, perforator flap, ulnar artery

## Abstract

An ulnar digital artery perforator flap was used for little finger reconstruction. The flap has a reliable blood supply, being perfused by a constant sizeable perforator. This paper describes a study of a cadaveric dissection with methylene blue dye that was conducted to prove the rationality and reliability of the blood supply. The position of the perforator is confirmed intraoperatively by an exploratory incision before committing to the distal incision. The flap used to cover the flexor aspect of the little finger in three cases yielded positive results. To our knowledge, a digital artery perforator flap of this nature is unprecedented. We propose to call this flap the B.J. Flap after our institute.

## INTRODUCTION

Soft tissue coverage of the palmer aspect of the little finger with considerations of colour and texture match and sensations has always been a challenge to the reconstructive surgeon. The options for resurfacing the flexor aspect of the little finger are limited. The cross-finger flap is generally the workhorse flap for little finger reconstruction across many centres.[1] The hypothenar area is used as a donor site very infrequently in clinical practice.[2–5] Distant pedicle flaps are bulky and may produce unaesthetic donor site deformities. Free flaps may not be accessible to all. In the present article, we put forth out preliminary report of the digital ulnar artery perforator flap for little finger reconstruction. It is a new flap based on the perforator of the ulnar digital artery used to resurface the little finger. The flap is thin and single stage and has minimal donor site morbidity, is technically simple and easily accessible.

### Surgical anatomy and operative technique

The skin over the hypothenar eminence is nourished by musculocutaneous or fasciocutaneous perforators through the hypothenar muscles or fascia. The distal half of the hypothenar eminence has a constant vascular participation from the ulnar palmer digital artery of the little finger.[6] Neural supply of this area is from the dorsal or the palmer cutaneous branches of the ulnar nerve.[6] The ulnar artery perforator flap is designed over the ulnar aspect of the hypothenar eminence, which is located over the abductor digiti minimi muscle. The flap is based on the perforator arising from the ulnar palmer digital artery of the little finger, which lies distal to the distal palmer crease.

After the defect is created, an exploratory incision is given over the palmar aspect of the hypothenar eminence overlying the abductor digiti minimi muscle. The perforators of the ulnar artery are visualized. There are, on average, three to four perforators that are seen. The distal most perforator is visualized. It lies 3–5 mm distal to the distal palmar crease. After the perforator is visualized, the distal margin of the flap is committed. Markings are performed as per planning in reverse. The flap can then be raised in a subfascial or suprafascial manner. Care is taken not to damage the ulnar neurovascular pedicle. The flap is then dissected up to the perforator and islanded on the perforator. The flap is rotated through 180° to cover the defect. The flap can be raised sufficiently large to cover the entire palmar aspect of the little finger up to the finger tip. The donor site is closed primarily or multiple Z plasties may be incorporated for the closure. The operative time is around 45 min to 1 hour. The finger is immobilized for 5–7 days, after which mobilization can be initiated.

The following points must be given due importance while flap harvesting:

1. Damage to the palmar digital artery must be avoided or else may result in ischaemia of the little finger.
2. The perforator on which the flap is based is a very tiny perforator and even a minimal amount of extra traction on the flap can lead to spasm of the perforator.
3. The perforator must always be kept moist by perfusing it with lignocaine solution.
4. Flap must be circumferentially dissected of all the fibrous strands so as to prevent kinking of the perforator. However, a rim of fat must be left surrounding the perforator.
5. Flap inset must be carried out without tension so as to avoid traction injury to the perforator.
6. To prevent traction to the perforator, the first two sutures are placed by the side of the perforator before suturing the distal end.
7. Dissection must always be performed under tourniquet control and magnification.

### Cadaver dissection and dye study

**Figure 1:**
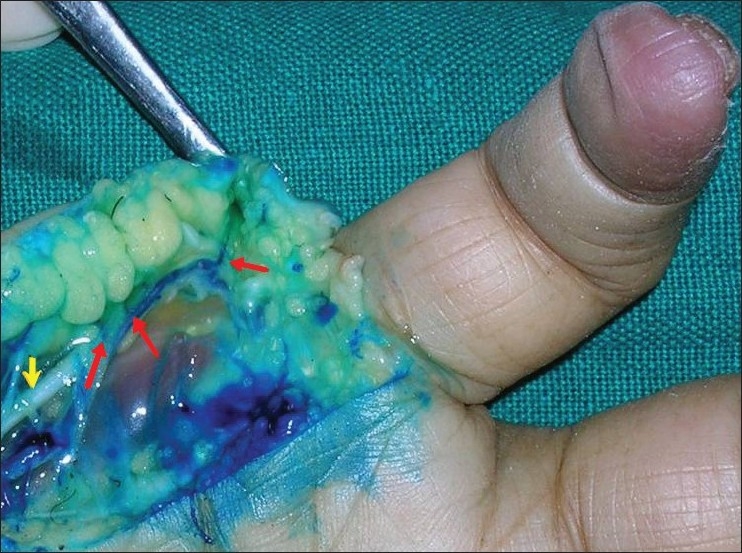
Dye study of the perforators. Red arrows showing the perforactors of the unlar artery, and yellow arrow demonstrating th ulnar digital nerve

**Figure 2:**
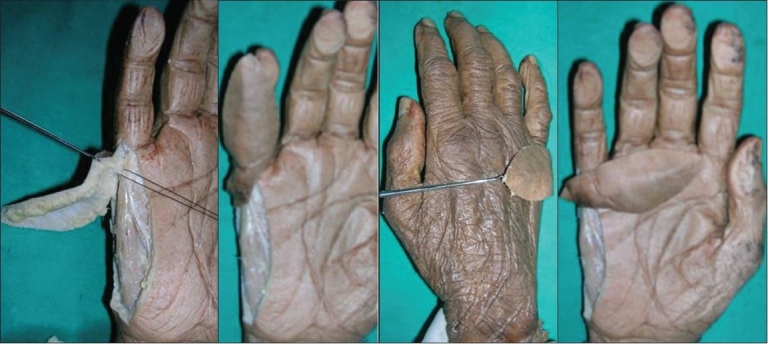
Cadaveric dissection showing the perforactor and arc of rotation of the ulnar artery perforator flap

Cadaveric dissection with dye study was performed in four limbs to study the anatomy, blood supply and arc of rotation of this flap [Figure 1]. Fifty per cent methylene blue dye (10 cc) was injected in the ulnar artery at the distal forearm level after ligating it proximally and the flap was dissected. Three to four perforators were seen to arise at regular intervals from the underlying digital artery to form a continuous longitudinal vascular arcade in the flap overlying the hypothenar eminence. The larger caliber perforators on visual inspection were those near the MCP joint, distal to the distal palmar crease [Figure 2].

## MATERIALS AND METHODS

Between November 2009 and March 2010, three patients were operated by the ulnar digital artery perforator flap. All the flaps were performed for post burn little finger contractures. All the flaps were used to cover the palmar aspect of the little finger where there was exposure of the tendons. There was one male (aged 45 years), one female (aged 32 years) and one male child (aged 11 years).

## RESULTS

**Figure 3:**
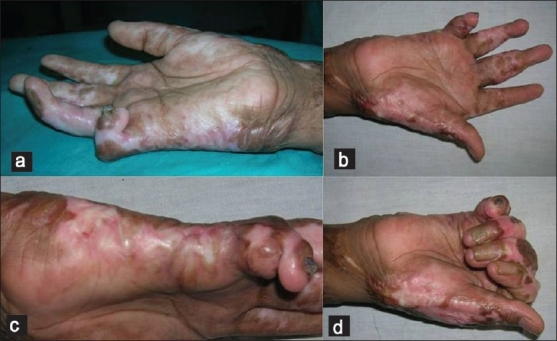
The ulnar artery perforator flap with donor site closed by Z plasty (a) Pre-operative (b) After contracture release and flap cover. (c) Donor site closure by multiple Z plasty (d) Restricted terminal flexion due to bulky flap

**Figure 4:**
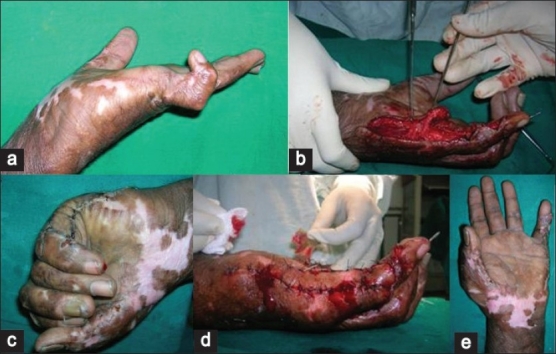
The ulnar artery perforator-based propellar flap (a) Pre-operative (b) distal perforator traumatized-flap islanded on proximal perforator (c) Postoperative (d). Flap propelled through 180 degrees to cover the defect and distal area Grafted (e) Post operative

**Figure 5:**
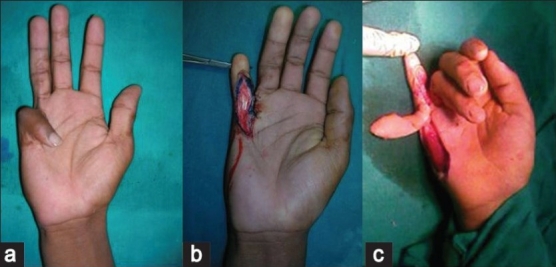
The ulnar artery perforator flap – suprafascial dissection (a) Pre-operative (b) Exposed tendons after contracture release. (c) Harvested ulnar artery perforator flap

**Figure 6:**
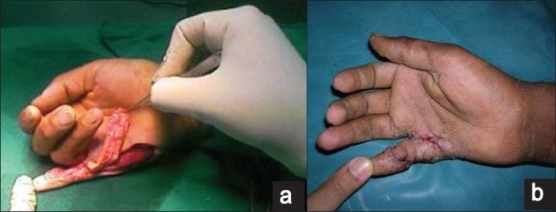
The ulnar artery perforator flap – suprafascial dissection (a) Suprafascial flap; can also be called sub dermal vascular network flap. (b) Incomplete extension of fi nger with well settled flap.

The follow-up ranged from 2 weeks to 20 weeks. The post operative course was uneventful. The first two flaps were raised in a subfascial manner and the third flap was raised in a suprafascial manner. There was minimal bulk in the flap in the first patient, which restricted few degrees of the terminal flexion; however, there was no functional deficit [Figure 3]. In the second patient, the distal most perforator was accidently damaged. The flap was then islanded on the proximal perforator and rotated through 180° to cover the defect like a propeller flap [Figure 4]. The distal area of the little finger over the distal interphalyngeal joint was grafted [Figure 4]. In the third patient, where a suprafascial flap was performed, there was problem in the vascularity of the finger on complete extension and the K wire had to be removed. There was some extension deficit in this patient [Figures 5 and 6]. However, there were no functional problems. The donor site was closed primarily in two patients and multiple Z plasties were performed in one patient. All the patients were satisfied with the flap procedure and the aesthetic outcome. There were no donor site problems. There was no painful scar observed at the donor site. The male child patient (aged 11 years; Figure 5a and b) had no pain and difficulty while writing with the operated right hand with the ulnar border resting. All the flaps were non innervated. No sensibility of the operated flaps was observed on early follow-up.

## DISCUSSION

We propose our preliminary report of the ulnar digital artery perforator flap for little finger reconstruction. To the best of our knowledge, this flap has not yet been reported.

Cross-finger flaps are commonly used for coverage of the flexor aspect of the little finger. However, it is a staged procedure with immobilization and potential for stiffness of the joint, and has a skin graft over the dorsum of the finger, which is not aesthetically acceptable to many.[1]

Gu *et al*.[3] used free flaps from the hypothenar region for resurfacing the hand defects with good results. Ueda and Inoue[4] reported a palmaris brevis musculocutaneous flap to reconstruct palmar loss of the thumb. However, use of free flaps is technically demanding and sacrifices the ulnar artery.

Shohei Omokawa *et al*.,[6] described the reverse hypothenar flap based on the ulnar pedicle. The arterial supply of this reverse flap is ensured by arterial communications between the radial and the ulnar palmar digital arteries of the little finger through dorsal or palmar arterial arcade. They did a pre operative digital Allens test. However, there is a potential for ischaemia of the little finger and cold intolerance after sacrificing the major vessel.

We propose the ulnar digital artery perforator flap for resurfacing the palmar aspect of the little finger. This flap is based on perforator of the ulnar digital vessel, thereby eliminating ischaemic problems and cold intolerance to the finger. It has a thin and durable fasciocutaneous component to cover the flexor aspect of the joint and a good colour and texture match as the local tissue is being used. The donor site is closed primarily and has a good aesthetic outcome.

It is a versatile flap, a single-stage procedure that can be used for post burn contracture defects as well as for traumatic defects, dupuytrens contractures and amputation stump coverage of the little finger. The flap harvest is easy and must be performed under tourniquet control and magnification. A rim of fat left along the perforator minimizes the chances of congestion in the post operative period. Although in our cases we have not performed a neurosensory flap, this flap can be harvested as a neurosensory flap with coaptation of the nerve distally.

The main limitation of this flap is a theoretical risk of a painful neuroma formation over the hypothenar donor area. However, in our few cases, there was no painful scar because the nerves were dissected out carefully and had a thick padding of subcutaneous fat. Shohei Omakawa *et al*.,[6] in their study of 11 patients of the reverse hypothenar flap found no donor site problems like painful scar or scar neuroma in their follow-up period of up to 97 months (average, 42 months). The cosmetic results in our patients were acceptable and there were no functional problems. In one patient, there was a potential for a linear band formation, where we carried out multiple Z plasties for closure of the defect.

As in all perforator flaps, there was minimal amount of congestion of the flap in the early post operative period, which settled down subsequently. However, in all our patients, there was persistent oedema of the flap even in the late post operative period.

One significant drawback of the procedure in case of subfascial flap was the bulkiness of the flap, resulting in restricted mobility of the little finger. This problem was overcome in our third case by a suprafascial dissection, thereby thinning the flap. Subsequent thinning of the flap can also be done, but it negates the advantage of the procedure being a single-stage procedure.

In case of hand burns, where there are multiple finger contractures and there is significant involvement of the dorsum of the fingers as well, cross-finger flap from the ring finger to cover the little finger is a difficult proposition. In these cases, the ulnar digital artery perforator flap can be considered as the flap of choice for little finger reconstruction.

## CONCLUSION

In our preliminary report of three cases, we describe a new flap for little finger reconstruction. We propose to call it the B.J. Flap after our institution. A more number of cases and a longer follow up is needed to establish this flap as one of the important options in little finger reconstruction. The ulnar artery perforator flap should be considered as one of the alternative options for resurfacing the little finger after traumatic loss of volar skin or after release of volar contracture of little finger.
